# Disclosing intimate partner violence to health care clinicians - What a difference the setting makes: A qualitative study

**DOI:** 10.1186/1471-2458-8-229

**Published:** 2008-07-04

**Authors:** Jane Liebschutz, Tracy Battaglia, Erin Finley, Tali Averbuch

**Affiliations:** 1 Section of General Internal Medicine, Department of Medicine, Boston Medical Center and Boston University School of Medicine, 801 Massachusetts Ave, Boston, MA 02118, USA; 2 Department of Social and Behavioral Sciences, Boston University School of Public Health, 801 Massachusetts Ave, Boston, MA 02118, USA; 3 Department of Anthropology, Emory University, 115 Dickey Dr, Atlanta, GA 30322, USA; 4 The Emory Center for Myth and Ritual in American Life, Emory University, 115 Dickey Dr, Atlanta, GA 30322, USA

## Abstract

**Background:**

Despite endorsement by national organizations, the impact of screening for intimate partner violence (IPV) is understudied, particularly as it occurs in different clinical settings. We analyzed interviews of IPV survivors to understand the risks and benefits of disclosing IPV to clinicians across specialties.

**Methods:**

Participants were English-speaking female IPV survivors recruited through IPV programs in Massachusetts. In-depth interviews describing medical encounters related to abuse were analyzed for common themes using Grounded Theory qualitative research methods. Encounters with health care clinicians were categorized by outcome (IPV disclosure by patient, discovery evidenced by discussion of IPV by clinician without patient disclosure, or non-disclosure), attribute (beneficial, unhelpful, harmful), and specialty (emergency department (ED), primary care (PC), obstetrics/gynecology (OB/GYN)).

**Results:**

Of 27 participants aged 18–56, 5 were white, 10 Latina, and 12 black. Of 59 relevant health care encounters, 23 were in ED, 17 in OB/GYN, and 19 in PC. Seven of 9 ED disclosures were characterized as unhelpful; the majority of disclosures in PC and OB/GYN were characterized as beneficial. There were no harmful disclosures in any setting. Unhelpful disclosures resulted in emotional distress and alienation from health care. Regardless of whether disclosure occurred, beneficial encounters were characterized by familiarity with the clinician, acknowledgement of the abuse, respect and relevant referrals.

**Conclusion:**

While no harms resulted from IPV disclosure, survivor satisfaction with disclosure is shaped by the setting of the encounter. Clinicians should aim to build a therapeutic relationship with IPV survivors that empowers and educates patients and does not demand disclosure.

## Background

The extensive physical and mental health burden of intimate partner violence (IPV) exposure has been documented in various settings [1-6]. In response, the medical community has prioritized IPV identification. In fact, the Joint Commission on Accreditation of Healthcare Organizations hospital standards call for IPV survivor identification and then referral to community services. In a complementary set of guidelines, the Family Violence Prevention Fund suggests clinicians inquire about IPV at every encounter for episodic care, such as Emergency Department visits, with higher case-finding rates as the measure of high quality [7].

Evidence to support IPV screening interventions includes surveys of patients who report expectations that a clinician inquire about IPV and increased satisfaction with the visit after being asked regardless of disclosure [8,9]. In a meta-analysis of the qualitative literature, Feder and colleagues found women IPV survivors value support and education whether or not they are ready to talk about the abuse, and consider most helpful relationships with physicians characterized by respectful support [10].

The most recent guidelines of the United States Preventive Services Task Force found insufficient evidence for screening for family violence due to lack of studies showing that a primary care based screening intervention helps reduce harmful outcomes [11]. In addition, the potential for negative outcomes of screening has not been examined [8,11-14]. Rather, the success of screening interventions tends to be measured in number of disclosures rather than in improvement of the survivor's overall condition [15-17]. Most studies of screening either lack a measure for the potential harm of disclosure or minimize such potential in written reports [15,17-19]. Although studies have shown higher disclosure in certain specialties [20], the difference in outcomes by specialty have not been well described. Rhodes and colleagues reported that many inquiries about IPV in an emergency room setting by physicians were perfunctory and did not lead to documentation or referral for other help [8,21].

Previously we reported results from a qualitative study of IPV survivors in which we examined those qualities of the patient-provider relationship that facilitate a safe and productive disclosure [22]. In that study, participants identified important provider characteristics, including: the ability to communicate a sense of personal concern; open communication; willingness to negotiate issues of control; confidentiality of medical information; shared decision-making; competency in medical care; careful listening; and taking ample time to address participant concerns [22]. Because of the reports of challenges to communication about IPV in emergency room settings and the emphasis on trust and communication in the patient-provider relationship, we hypothesized that the setting of disclosure of IPV might be important to the patient experience. Furthermore, such differences might inform clinical practice in varied medical settings.

In this paper we present the results of a re-analysis of participants' descriptions of patient-provider encounters to examine potential harms and benefits of IPV disclosure. We explored whether the specialty of care was related to the outcomes of disclosure, and identified a series of factors affecting these outcomes across primary care, obstetrics/gynecology and emergency department specialties.

## Methods

### Study Design

Ethnographic interviewing elicited IPV survivors' experiences interacting with both physician and non-physician health care providers. Grounded theory, a method of qualitative analysis [23], was used to elucidate views on patient-provider encounters revealed in the narrative data.

### Study Participants

Twenty-seven IPV survivors were recruited from community-based domestic violence counseling or sheltering programs in eastern Massachusetts. They were recruited either through referral by local shelter staff or through a flier sent to all domestic violence programs in eastern Massachusetts. Eligible participants were female, ages 18 to 64, English-speaking, with a history of an abusive intimate partner relationship within the past 3 years. Each participant provided written informed consent and was compensated $25.

### Interview Technique

After approval by the Boston University Medical Center Institutional Review Board, data were collected from October 1996 through November 2000. Open-ended, in-depth interviews, conducted by 1 of 2 authors (JL, TB), both primary care physicians, were audio-taped and lasted 1–2 hours.

Using an interview guide, the interviewer asked participants to describe encounters with health care clinicians both related and unrelated to the abusive relationship after the onset of the abuse. While most participants related to the onset of the adult intimate partner violence, others spontaneously mentioned experiences with healthcare providers during adolescence or relating to childhood abuse. The participants were asked to provide information on perceived barriers to care and the abusive relationship over the past three years. Interviews were iterative; participants enrolled later in the data collection interval were questioned about themes revealed in previous interviews.

### Analysis

Each audio-taped interview was transcribed verbatim by a professional transcriber, reviewed for accuracy and de-identified. Authors independently reviewed transcripts to identify common themes which were developed into a preliminary coding scheme with the first 10 interviews. An advisory group of domestic violence advocates and survivors helped revise this scheme and suggest new concepts. The authors then independently coded the interviews using this revised coding scheme. Coding was compared and differences of opinion resolved through examination of the text.

Using NUD*ST qualitative research software (QSR International, Pty., Ltd., Melbourne, Australia) for data organization and coding, separate narratives representing a single patient-clinician relationship were identified and labeled as *encounters*. Encounters, which could be composed of a single interaction or continued contact over a period of years, were first categorized into "related to abuse" or "unrelated to abuse". As we did reiterative coding and analysis to understand the specific effect of disclosing (or not-disclosing) IPV, these unrelated encounters did not offer relevant material to allow categorization into a specific outcome (disclosure, discovery/discussion, nondisclosure) and were thus dropped from the analyses. Each medical encounter related to abuse was then coded according to three characteristics: outcome, specialty and attribute.

The first of these, outcome, described three mutually exclusive types of encounters: disclosure, discovery, and non-disclosure. A disclosure occurred when a participant reported telling her clinician about IPV. When a participant perceived her clinician knew of the abuse when she had not made an explicit disclosure, the outcome was labeled discovery. To be labeled discovery, the participants made explicit reference that the provider discussed some aspect of IPV, such as counseling or referral, even without explicit disclosure of IPV. All other encounters that did not fall into disclosure or discovery were labeled non-disclosure. To qualify for non-disclosure, one of two circumstances had to apply. First, the provider asked but the participant purposely did not disclose. Second, the participant was in an actively abusive relationship but did not spontaneously disclose, such as during treatment for injury, or during medical or pregnancy related care.

Each encounter was also coded for its *specialty*: Emergency Department (ED), Obstetrical or Gynecological Care (OB/GYN), Primary Care (PC) or other. PC included pediatricians and family physicians identified as the primary care provider but who may have also provided obstetrical care. Encounters occurring in other specialties (e.g. mental health, surgery) were excluded from this analysis because there were too few of any single type.

The final category, attribute, described the participant's level of satisfaction with the encounter as a result of whether she perceived the interaction as beneficial, harmful or unhelpful. For example, if an unpleasant interaction ended in the participant accepting help or receiving information that she found useful, we labeled it beneficial. Harmful interactions were ones resulting in injury to self, child or direct worsening of abuse. We classified negative reports not resulting in actual harm as unhelpful. When we were unable to categorize attribute due to a lack of information or contradictory descriptions, we excluded that encounter from analysis. Finally, we conducted a comparative analysis to explore the characteristics of encounters across outcomes, specialties and attributes.

## Results

We interviewed 27 women; 12 were black, 10 Latina, and 5 White. Fourteen were recruited by domestic violence staff, and thirteen contacted the authors in response to the informational flier. Sixteen were living in a residential program at the time of the interview. Participant ages ranged from 18–56 years; median age was 31 years. Twenty-three participants had at least one child.

**Figure 1:**
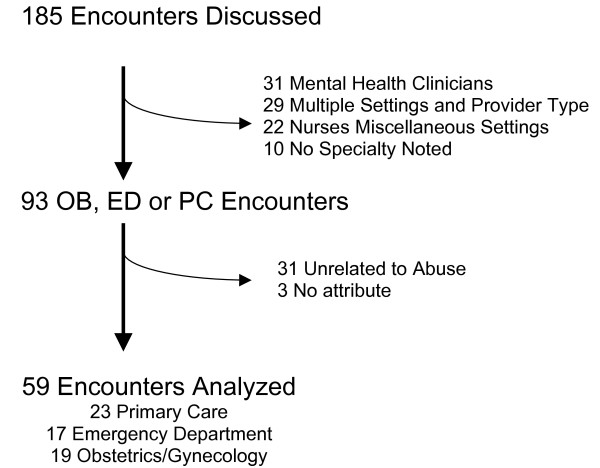
Encounter Classification Flow Chart.

A total of 185 health care encounters were described. The number of encounters per participant ranged from 3–12; median number of encounters was 7. Although it was frequently difficult to determine the professional designation (physician, nurse, therapist) of an individual provider, specialty was clear in 175 encounters. The thirty-one mental health encounters were excluded because most were visits specifically related to the IPV. Twenty-two were nurses from different treatment settings (inpatient, public health, etc.). Of the twenty-nine other encounters, there were two few (<5) of any single type and could not be easily combined into categories- such as radiology technicians, surgeons, ambulance drivers, physical therapist, child protective service worker, medical subspecialist, etc. Thirty-one were excluded because they were unrelated to abuse, and did not contribute to the analysis presented in this paper, the impact of IPV disclosure. Another three were unable to be classified by attribute, leaving a sample pool of 59 encounters (23 primary care, 17 ED, 19 OB/GYN) representing 25 participants (Figure 1).

**Figure 2:**
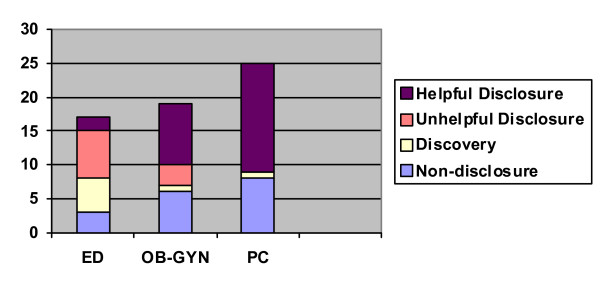
Encounter Attribute by Specialty.

Thirty-five (59%) of these encounters involved IPV disclosure to the clinician, 7 (12%) in discovery, and 17 (29%) in non-disclosure. Of the disclosures, 25 (71%) were beneficial. Among discoveries, 4 were beneficial (57%), while among non-disclosures, 6 (35%) were beneficial. Setting of care was associated with reported satisfaction from disclosure. In the ED, 2 (22%) disclosures were beneficial. Of OB/GYN disclosures, 9 (75%) were beneficial. In primary care, all 14 disclosures were beneficial (Figure 2). There were no harmful disclosures in any specialty, and the remaining disclosures were unhelpful. We discuss these findings further in the paragraphs below.

### Consequences of Unhelpful Disclosures: Fear and Avoidance of Healthcare

The most serious negative consequences of disclosure occurred in two participants who reported feeling endangered because of the disclosure, both after treatment for acute injuries in the ED. However, neither experienced any actual increase in violence. In two OB/GYN visits, participants found disclosure experiences so problematic they ultimately left their providers. The remaining 5 unhelpful disclosure experiences resulted in dissatisfaction without cessation of the clinical relationship.

Several participants were concerned by practitioners' tendency to encourage extreme "solutions" to the violence, like telling women to file a police report immediately. While in the ED, one participant reported being told, "Just tell me the name and where he's at and we'll send the police at him right now." She recalled thinking: "But what makes them think he won't come back and kill me?" The participant did not contact the police, and returned home in fear.

A number of participants indicated the cumulative effect of unhelpful disclosure experiences was avoidance of health care encounters. One participant noted:

"*I used to go without medical treatment... I'd wait until it wasn't a choice anymore. And I'd wind up having to go to the emergency room.*"

Another participant revealed:

"*Somebody would find out something was happening in my house, like a social worker, a doctor, a nurse or whatever, I would stop going there and go somewhere else.*"

The lack of an emotional connection with the clinician was a prominent feature in participants' discussion of unhelpful disclosures. Describing an ED visit, one participant commented:

"*He checked me, he didn't ask any questions, nothing, and they took x-rays and pulled out of there... Maybe I was hoping... that they would talk to me? I mean, they checked me out... but I didn't feel like... emotionally? Like maybe talk, some kind of comfort?*"

The lack of effective communication on safety assessment, referrals, and follow-up for IPV was also a consistent problem in unhelpful disclosures. During her prenatal intake visit, for example, one participant disclosed ongoing violence, the name of her abusive partner, and his status as an undocumented foreigner. Not understanding what follow-up would occur, she became petrified that her husband would assault her for revealing his status. She subsequently switched to another prenatal care provider, where she lied about her home life. Several participants disclosed abuse but reported receiving little helpful advice from the clinician. The woman encouraged to contact the police above did not remember being offered contact information for safe houses in the area. Similarly, after treatment for acute injuries, one participant reported: "I don't recall ever getting information about a shelter... or an advocate speaking to me. Any of that."

### Benefits of Disclosure: Making Changes, Improving Self-Esteem, Building Relationships

Eleven of the 25 beneficial disclosure experiences led directly to a change in the participant's circumstances, such as leaving the abusive spouse, entering a detoxification program or filing a police report. For example, one participant with newborn twins and a toddler reported that after disclosing her husband's assaults,

"*I started off in a shelter in* [a distant town] *because I couldn't find one that would take all of us right away... My* [OB] *got on the phone with Social Service to try and get me all the help that I needed.*"

In others, changes resulted after a clinician worked with a participant over a period of time. One participant described the effect of the close relationship she developed with her midwife:

"*She was real supportive through my pregnancy, and told me 'everything will be okay,' and I'll be a good mother. And I am a good mother. 'Cause it made me realize a lot of things... that I was thinkin', and I had my whole life to live, but now I could do better with myself, as well as with my daughter... I'm workin' on gettin' housin', takin' care of my schoolin', just bein' responsible.*"

Instead of an immediate end to the abuse, these patient-clinician encounters resulted in a shift in the participant's self-esteem, perception of the violent relationship, or awareness of alternatives, eventually empowering her to seek help for the abuse on her own. For example, clinicians' assurances that relationship violence was unacceptable resonated with several participants. One participant reported her primary care doctor's sympathetic insistence that the batterer's behavior was wrong set the stage for her to take action:

"*She was like, 'No... no one who loves you will put their hands on you.' You know, it's not right. 'That's not real love.'...After* [he broke] *the wrist, I said, 'No more.'*"

After being treated in the ED for IPV-related injuries, another participant left with information on local safe houses that she later consulted when she was ready to leave her abuser.

Some of the beneficial disclosure experiences resulted in a more positive attitude toward health care in general, as in 5 instances where participants reported feeling a greater closeness with their clinicians despite no other change in their circumstances. Whether or not disclosure led to change, analysis revealed three common characteristics of provider behavior in beneficial disclosures: 1) explicit acknowledgement of the content of the disclosure (all cases), 2) demonstration of a caring attitude after disclosure (most cases) and 3) specific referral to other resources (some cases). For example, one participant said an ED clinician explicitly acknowledged her abuse and demonstrated concern:

"*He said, well, 'I hear you're in a battered women's shelter. What's the deal? I take a special interest in domestic violence and what happens,' and he sat and talked to me. I felt comfortable in talking to him because he was showing this special interest in what was going on with me.*"

Also of note, in all but two beneficial disclosures the participant reported familiarity with the clinician. In primary care, these relationships involved getting to know the clinician through a variety of contacts both related and unrelated to the IPV. In OB/GYN, these relationships generally formed during prenatal care, or in the peri-partum period when the participant had daily contact with hospital clinicians. Such familiarity can also occur in the ED setting, as in one case where the participant accepted advice from a nurse who had treated her a few weeks earlier for IPV-related injuries. When the participant returned to the ED with more injuries, the nurse recognized her:

"*And I started crying, and she's like, 'Two weeks ago you was here, now you're back here again today and it's for the same thing. Your face isn't all bruised up like it was two weeks ago, but you're hurtin'. What's goin' on?' I broke down and told her...She was like, 'Well, you don't need to be in a relationship like that.*"'

The participant acted on referrals and left her abusive partner as a result of this encounter.

### Potential Benefits and Problems without Disclosure

The common thread to benefits and problems without verbal disclosure by the participant included explicit clinician acknowledgement of potential abuse (or lack thereof). In particular, participants reported being upset by health care providers who they felt should have recognized IPV but did not acknowledge it. This, in turn, led to avoidance of healthcare. One participant reported that healthcare personnel failed to bring up IPV even after her husband yelled at her in the ED during two separate visits. She interpreted this lack of acknowledgement as an indication that clinicians did not care to get more involved. Another participant was particularly disappointed that her primary care clinician did not address the abuse with her, given that she had received counseling about it from his nursing staff: "He never gave me any type of indication...he didn't talk to me about it. That's why I left him...because he wasn't really direct with me."

Several participants reported benefit when the clinician spoke openly with the participant about IPV but did not insist upon disclosure. Furthermore, clinicians in these encounters used verbal and non-verbal cues to convey concern, and offered options for intervention while not forcing the participant to take action. The aftermath of acute injury was a particularly vulnerable time, as survivors were emotionally and physically exhausted as well as fearful of more injury.

"*They asked me, 'How did it happen?' 'What happened to you?' 'Who did that?' I was in so much pain that I really didn't want to talk about it.*"

A critical component of beneficial non-disclosure experiences was consideration of the patient's safety, as in this ED visit:

"*She realized that I had other bruises on me. I thought he might hear her and I was like, 'No. Let's just drop the conversation. Let's just get me stitched up.' My husband came in so there was no more talk about it. When I left, she called me apart, and she* [said]: *'you could call here in an emergency and we could get you some help.*"'

Another example included ED staff suggestion that a participant treated for acute injuries continue care in PC: "and they gave me a choice, 'would you rather go to your doctor and tell them what happened?"' As a result of that referral, she revealed the abuse to her primary care clinician.

## Discussion

Narratives of intimate partner violence survivors reveal no actual harms occurred as a result of disclosure of abuse to health care clinicians. However, some negative disclosure experiences did impair subsequent interactions with the health care provider as well as increase emotional distress. The benefits included immediate changes (e.g. filing a restraining order), improvement in self-esteem to facilitate long term changes, and relationship building with health care clinicians. The setting of care appeared to influence these outcomes, impacted strongly by patient familiarity with the clinician.

This study reinforces insights from prior studies that asking about IPV in longitudinal care specialties offers the greatest opportunity for disclosure [20]. Indeed, participants valued clinicians who knew them well over time and were thus more likely to find disclosure in such settings beneficial. The benefits of disclosure reported here went beyond simply providing information, as might have been expected, but suggest an impact on patient self-worth and empowerment. This suggests that the relationship between clinician and patient can itself be a point of healing, and should reassure clinicians that extensive training in domestic violence or counseling is not as important as nurturing the relationship with a patient [24,25].

In all specialties, participants were more likely to disclose IPV and find disclosure beneficial if clinicians respectfully addressed the abuse, ensured participants' physical safety after an assault, assured participants of confidentiality regarding disclosed information, gave patient choices for action and demonstrated emotional support. Indeed, our study demonstrates that inquiry and discussion of IPV in the right setting can be a powerful tool for change.

Despite the increased potential to identify and refer a victim of IPV in the aftermath of an acute injury [26,27], participants in this study had mixed experiences with disclosure in the ED. Someone being treated for an acute injury as a direct result of IPV is likely to be in a highly charged state from the physical and emotional pain [28]. These women may feel particularly vulnerable and sensitive to any perceived failure of empathy on the part of the clinician. Furthermore, the probability of a beneficial disclosure in the ED may be lower with lack of familiarity with the clinician, a key element in many helpful disclosures. There may also be organizational barriers [29], such as lack of stretches of time to spend with any one patient while trying to manage an emergency department with multiple patients with differing levels of acuity. Rhodes's analysis of audio-taped encounters between physicians and IPV survivors confirms the difficulty exhibited by many clinicians' attempting to address this issue [21]. Thus, the ability to process and receive help related to IPV may be higher if it is done outside the context of emergency care. Treatments for acute injury related to IPV should also be viewed by clinicians as opportunities to educate and empower the patient, leaving her with options to exercise when she is ready. This may empower clinicians as well if they feel they have a task in helping the patient rather than just uncovering a painful problem. Because ongoing relationships were more likely to lead to helpful disclosure experiences in this study, acute care providers should, with the patient's permission, inform her regular clinician of her visit [30].

IPV case-finding may satisfy the need for a quantifiable, appropriate quality improvement measure. However, measuring case finding alone may obscure whether the inquiry is occurring in an empowering and safe manner that benefits survivors. In settings such as the ED or even inpatient hospital care, where the risks of disclosure may be higher, other measures of quality could include surveys of patients at high risk for IPV to assess whether they received any education about resources or options for IPV. Future studies of intervention for IPV could consider measuring empowerment and trust around IPV disclosure in the health care setting. Outcome measures often determine the emphasis of clinical care [31,32]. If an organization such as the Joint Commission chose a process measure of IPV education and patient empowerment, it might spur clinical practice to change.

There are several limitations to this study. First, we were not always able to determine the exact nature of the visit or specialty. Furthermore, participants were not directly asked to compare their experiences; differences were gleaned from the stories they told. This is typical of qualitative research studies in which unexpected themes emerge from close examination of the data. Self-report is subject to recall bias, which may be particularly affected by any post-traumatic stress disorder related to abuse. The interviews occurred almost 10 years ago and clinician response might have improved since then, given the educational efforts with medical students and residents. However, this has not been demonstrated in more recent studies [21]. Finally, we only interviewed women who had used community resources and may not represent all IPV survivors.

## Conclusion

Our results reveal that whether or not disclosure of abuse is achieved, clinician conversations with survivors about IPV have a powerful impact on both positive and negative outcomes. When these conversations occur in the context of a supportive relationship with that clinician, positive outcomes are more likely. Although these findings will need to be replicated in other settings, this study suggests a need to tailor interventions for women who experience IPV to the nature of the clinical specialty, particularly treatment of acute injury. Our findings indicate that it is not enough for health care providers to simply ask about abuse. Clinicians should aim for a therapeutic relationship with IPV survivors that does not demand disclosure or action, but instead empowers and educates the patient.

## Competing interests

The authors declare that they have no competing interests.

## Authors' contributions

JL designed the study. JL and TB conducted the interviews. All coauthors helped analyze data and reviewed the manuscript drafted by JL for important intellectual content. All coauthors approved the final draft.

## Pre-publication history

The pre-publication history for this paper can be accessed here:


